# Validation of α‐Synuclein in L1CAM‐Immunocaptured Exosomes as a Biomarker for the Stratification of Parkinsonian Syndromes

**DOI:** 10.1002/mds.28591

**Published:** 2021-04-07

**Authors:** Cheng Jiang, Franziska Hopfner, Daniela Berg, Michele T. Hu, Andrea Pilotto, Barbara Borroni, Jason J. Davis, George K. Tofaris

**Affiliations:** ^1^ Nuffield Department of Clinical Neurosciences John Radcliffe Hospital, University of Oxford Oxford UK; ^2^ Department of Neurology Christian‐Albrechts‐University Kiel Kiel Germany; ^3^ Department of Clinical and Experimental Sciences, Neurology Unit University of Brescia Brescia Italy; ^4^ Department of Chemistry, Physical and Theoretical Chemistry Laboratory University of Oxford Oxford UK

**Keywords:** neurodegeneration, L1CAM, biomarker, extracellular vesicles, synuclein

## Abstract

**Background:**

Parkinson's disease is characterized by intraneuronal α‐synuclein aggregation. Currently there is no α‐synuclein‐based blood test in clinical practice.

**Objectives:**

Our aim was to assess by means of further testing and analysis whether α‐synuclein measurements in serum L1CAM‐immunocaptured exosomes can differentiate Parkinson's disease from related movement disorders.

**Methods:**

We used poly(carboxybetaine‐methacrylate)‐coated magnetic beads to isolate L1CAM‐positive exosomes and triplexed electrochemiluminescence to measure exosomal α‐synuclein, clusterin, and syntenin‐1 from 267 serum samples. Combined analysis of our current and previously published data from the Oxford, Kiel, Brescia, and PROSPECT cohorts consisting of individuals (total n = 735) with Parkinson's disease (n = 290), multiple system atrophy (MSA, n = 50), progressive supranuclear palsy (n = 116), corticobasal syndrome (n = 88), and healthy controls (n = 191) was done using 2‐stage (training vs validation) receiver operating characteristic analysis.

**Results:**

We established that α‐synuclein level in L1CAM‐immunocaptured exosomes above 14 pg/mL is a robust biomarker across cohorts that distinguishes Parkinson's disease from MSA (AUC, 0.90 vs 0.98) or 4‐repeat tauopathies (AUC, 0.93 vs 0.94). We confirmed that exosomal clusterin is elevated in subjects with 4‐repeat tauopathy, and when combined with α‐synuclein, it improved the performance of the assay in differentiating Parkinson's disease from 4‐repeat tauopathies to AUC, 0.98 versus 0.99. Correction for the generic exosomal protein syntenin‐1 did not consistently improve the performance of the assay.

**Conclusions:**

α‐Synuclein and clusterin in L1CAM‐immunocaptured serum exosomes is a validated blood test for the molecular stratification of neuronal α‐synucleinopathy (ie, Lewy body pathology) versus phenotypically related neurodegenerative movement disorders. © 2021 The Authors. *Movement Disorders* published by Wiley Periodicals LLC on behalf of International Parkinson and Movement Disorder Society

## Graphical abstract

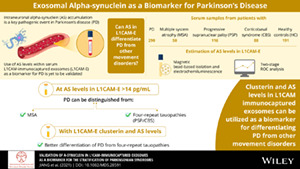

November Infographic 2 : Validation of neuron‐derived exosomal a‐synuclein in the stratification of Parkinsonian syndromes

Parkinson's disease (PD) is the commonest movement disorder characterized by intraneuronal α‐synuclein aggregation into Lewy bodies or neurites. In contrast, predominantly oligodendroglial α‐synuclein accumulation is the primary neuropathological feature of multiple system atrophy (MSA), a life‐limiting condition characterized by extrapyramidal, cerebellar, and autonomic symptoms.^1^, ^2^ Although the diagnostic accuracy for idiopathic PD is high in a specialist movement disorders clinic,^3^ this is not always the case in the early stages of the disease, when atypical parkinsonism, principally MSA and sometimes progressive supranuclear gaze palsy (PSP) or corticobasal syndrome (CBS) can mimic idiopathic PD.^4^ Previous clinicopathological studies showed that approximately 30% of patients with pathologically proven MSA seen in movement disorders clinics and up to 50% of cases diagnosed in a general neurology clinic carry the incorrect diagnosis at death.^5^, ^6^, ^7^

We have recently reported that the content of α‐synuclein in L1CAM‐immunocaptured serum exosomes is elevated from the prodromal phase of PD and could be used in the differential diagnosis of neuronal α‐synuclein pathology seen in PD from other proteionopathies.^8^ Here we aimed to validate our earlier findings using additional samples from the Kiel PD cohort^9^ (n = 60) and the PROSPECT cohort^10^ (n = 207), which is a UK‐wide prospective cohort of patients with atypical parkinsonian syndromes and controls, followed by combined analysis with our previous data,^8^ comprising a total of 4 cohorts consisting of patients with PD (n = 290), MSA (n = 50), PSP (n = 116), and CBS (n = 88) and healthy controls (n = 191). To the best of our knowledge, this constitutes the largest study of exosomal α‐synuclein in PD and related disorders that tested and confirmed specific cutoff measurements for the molecular stratification of parkinsonian syndromes.

## 1 Methods

### 1.1 Patient Populations

A total of 735 subjects were included in this study (Table 1). Serum samples and clinical data were collected from patients with PD (n = 290), MSA (n = 50), PSP (n = 116), and CBS (n = 88) and healthy controls (HC, n = 191). Patients and controls were recruited from 4 centers: the Oxford Parkinson's Disease Discovery cohort,^11^ the Kiel‐PD cohort,^9^ the Brescia cohort,^12^ and the UK‐wide PROSPECT cohort, which consists of 7 UK study sites (University College London, Oxford, Cambridge, Newcastle, Brighton, Newport, and Manchester).^10^ Diagnostic criteria were uniformly applied across cohorts for PD,^13^ MSA,^14^ probable PSP,^15^ and CBS.^16^ Healthy controls were recruited from patients’ partners without a history of neurological or psychiatric disease or family history of PD.

**TABLE 1:** Summary of demographics, clinical scores, and biomarker concentrations (mean ± SD) from the 4 cohorts used in this study

Sites		PD	MSA	HC	PSP	CBS
Oxford	Number of individuals	48	14	31	—	—
	Male/female	36/12	10/4	22/9	—	—
	Age	62·8 ± 9.3	68.1 ± 10.8	66.3 ± 8.8	—	—
	Duration of disease (years)	5.4 ± 3.4	4.9 ± 2.6	na	—	—
	UPDRS	32.9	27.7	na	—	—
	MoCA	27.1	27.3	na	—	—
	exo α‐Synuclein (pg/mL)	22.4 ± 9.5	10.7 ± 4.5	12.5 ± 5.1	—	—
	exo Clusterin (ng/mL)	7.9 ± 3.6	6.8 ± 3.2	11.3 ± 2.6	—	—
	exo Syntenin‐1 (ng/mL)	43.3 ± 21.6	14.8 ± 5.8	33.4 ± 15.9	—	—
Brescia	Number of individuals	27	—	—	35	45
	Male/female	17/10	—	—	18/17	27/18
	Age	65.0 ± 9.4	—	—	68.0 ± 7.5	61.1 ± 7.2
	Duration of disease (years)	na	—	—	2.8 ± 1.8	1.9 ± 1.3
	UPDRS	20.1	—	—	24.5	22.3
	MoCA	26.8	—	—	21.4	22.5
	exo α‐Synuclein (pg/mL)	25.6 ± 19	—	—	9.2 ± 4.9	9.93 ± 3.7
	exo Clusterin (ng/mL)	7.6 ± 5.8	—	—	18.4 ± 8.8	16.2 ± 6.1
	exo Syntenin‐1 (ng/mL)	23.0 ± 10	—	—	44.3 ± 23	54.7 ± 25
Kiel	Number of individuals	215	—	113	—	—
	Male/female	136/79	—	72/41	—	—
	Age	67.6 ± 4.8	—	59.0 ± 4.8	—	—
	Duration of disease (years)	9.4 ± 2.8	—	na	—	—
	UPDRS	24.6	—	na	—	—
	MoCA	26.5	—	na	—	—
	exo α‐Synuclein (pg/mL)	30.1 ± 17.9	—	12.7 ± 6.1	—	—
	exo Clusterin (ng/mL)	11.3 ± 6.3	—	8.1 ± 5.2	—	—
	exo Syntenin‐1 (ng/mL)	22.7 ± 13.5	—	18.8 ± 12.0	—	—
PROSPECT	Number of individuals	—	36	47	81	43
	Male/female	—	25/11	17/30	54/27	14/29
	Age	—	66.1 ± 9.1	68.0 ± 6.8	71.0 ± 6.4	68.2 ± 7.1
	Duration of disease (years)	—	5.6 ± 2.7	na	4.2 ± 2.5	4.7 ± 2.7
	UPDRS	—			37.6	49.9
	MoCA	—	24.8	27.1	22.7	19.3
	exo α‐Synuclein (pg/mL)	—	8.7 ± 2.8	10.8 ± 4.5	11.1 ± 2.9	11.4 ± 3.9
	exo Clusterin (ng/mL)	—	12.2 ± 7.0	13.0 ± 5.2	20.0 ± 7.2	21.2 ± 9.6
	exo Syntenin‐1 (ng/mL)	—	16.9 ± 6.0	24.5 ± 13.6	39.6 ± 15.7	37.7 ± 15.2

PD, Parkinson's disease; MSA, multiple system atrophy; HC, healthy controls; PSP, progressive supranuclear gaze palsy; CBS, corticobasal syndrome; UPDRS, Unified Parkinson's Disease Rating Scale; MoCA, Montreal Cognitive Assessment; na, not applicable.

### 1.2 Ethical Approval

Written informed consent was obtained from all participants. These patient studies were approved by the corresponding local hospital ethics committee: the Oxford Discovery cohort by the South Central Oxford A Research Ethics Committee (IRAS 188167), the PROSPECT cohort by the London‐Queen Square Research Ethics Committee (IRAS 149695), the Kiel‐PD cohort by the Ethics Committee of Kiel University (AZ:D:477/11), and the Brescia cohort by the Spedali Civili Hospital Ethics Committee (NP1471 and NP2189).

### 1.3 Exosome Immunocapture

Our isolation protocol and its validation followed MISEV2018 recommendations as described in Jiang et al.^8^ Briefly, blood samples from the 4 centers were collected during patient assessment, the serum was isolated by centrifugation at 2000*g* for 10 minutes, aliquoted, and frozen at −80°C until further use. All samples were sent on dry ice, blindly coded, and processed in Oxford. All samples went only 1 freeze–thaw cycle. For exosome isolation, a sequential spin (300*g* for 10 minutes, 2000*g* for 20 minutes, and 10 000*g* for 30 minutes) was used to remove cellular debris, protein aggregates, and fatty material in the serum. The 0.25 mL of supernatant, that is, precleared serum, was transferred to protein low‐binding tubes (Eppendorf) for immunocapture using anti‐L1CAM antibodies (ab80832, Abcam) preconjugated to poly(carboxybetaine methacrylate)‐coated beads that reduce nonspecific adsorption.^17^ The immunobeads were incubated at 4°C overnight on a rotating mixer, and bead‐exosome complexes were collected by a magnetic rack and washed successively with 0.05% Tween‐20 in phosphate‐buffered saline and phosphate‐buffered saline (PBS). Isolated exosomes were lysed in 50 μL of 1% triton X‐100 in PBS containing 4% protease inhibitors for 15 minutes at room temperature, and the lysate was collected for exosomal protein quantification after magnetic separation of the beads. Exosomes were extracted in batches with disease and control samples distributed into each batch followed by sample blinding.

### 1.4 Detection of Exosomal Proteins

Electrochemiluminescence (ECL) was performed in 96‐well Meso Scale Discovery (MSD) U‐Plex plates that enable multiplexing of markers in the same exosome preparation. All steps were performed at room temperature. Three unique linkers for the selected markers (syntenin‐1, clusterin, and α‐synuclein) were used according to the manufacturer's protocol. After 3 washes, detection antibodies with Sulfo‐TAG labeling were incubated for 1 hour. Following washes and the addition of MSD Read buffer, the plates were read using the MSD‐ECL platform (QuickPlex SQ 120), and data were analyzed with the MSD Discovery Workbench 3.0 Data Analysis Toolbox. Antibody pairs for clusterin and α‐synuclein were provided by MSD and preconjugated with biotin and ruthenium tag. Additive‐free anti‐syntenin‐1 goat polyclonal antibody (PAB7132, Abnova) and anti‐syntenin‐1 rabbit monoclonal antibody (ab236071, Abcam) were conjugated with biotin and ruthenium and used as capture and detection antibodies, respectively. For combined exosomal α‐synuclein, clusterin, and syntenin‐1, we used a triplex MSD that we previously demonstrated to specifically detect these markers in immunocaptured exosomes.^8^

### 1.5 Statistical Analysis

For multiple comparisons we performed nonparametric statistical testing, as the data were not normally distributed (Kruskal–Wallis 1‐way analysis of variance with the Dunn test for post hoc comparison between individual pairings) using Prism 8 Graphpad (San Diego, CA). Relationships between exosome markers and disease duration, MoCA score, and UPDRS motor score were analyzed with bivariate correlation using Pearson's correlation coefficients. To assess the performance of the biomarker across cohorts, we used a 2‐stage model with 2 cohorts used as training and 2 separate cohorts for validation. Data from these groups were analyzed using receiver operating characteristics. The “optimum” cutoff point was determined by Youden's index, that is, the value associated with the maximal value of sensitivity + specificity − 1. Values with *P* < 0.05 were regarded as significant. Logistic regression analysis was used to determine the best combination of different protein markers (clusterin and α‐synuclein) for discriminating between diagnostic groups or sets of subgroups. The robust regression and outlier removal method was applied to test for outliers.

### 1.6 Data Availability

Anonymized individual participant data and the study protocol will be shared with qualified parties on request to the corresponding author.

## 2 Results

We previously developed and extensively validated an assay for the immunocapture of L1CAM‐positive exosomes from serum using polymer‐coated antifouling magnetic beads that reduce non‐specific binding of free α‐synuclein and other abundant serum proteins.^8^, ^17^ Using this method we blindly isolated L1CAM‐positive exosomes from 250 μL of serum from patients with PD (n = 60), MSA (n = 36), PSP (n = 81), and CBS (n = 43) and from HC (n = 47). We then used a triplex MSD detection platform that we previously established^8^ to measure the content of α‐synuclein, clusterin, and syntenin‐1 in the isolated L1CAM‐positive exosomes. First, we confirmed (data shown as mean ± SD) that α‐synuclein content in L1CAM‐positive exosomes is elevated in PD (22.5 ± 4.8 pg exo α‐synuclein/mL serum) by ~2‐fold when compared with controls (10.8 ± 4.5 pg exo α‐synuclein/mL serum), MSA (8.7 ± 2.8 pg exo α‐synuclein/mL serum), or other tauopathies (11.3 ± 3.4 pg exo α‐synuclein/mL serum), as shown in Figure S1A. Second, we confirmed our previous observation that exosomal (exo) clusterin is elevated in PSP (20.0 ± 7.2 ng exo clusterin/mL serum) and CBS (21.2 ± 9.6 ng exo clusterin/mL serum), compared with PD (12.1 ± 4.8 ng exo clusterin/mL serum) or HC (13.0 ± 5.2 ng exo clusterin/mL serum), as shown in Figure S1B. Syntenin‐1, which is a generic exosome cargo protein, was not consistently elevated in any disease (Fig. S1C).

**FIG. 1:**
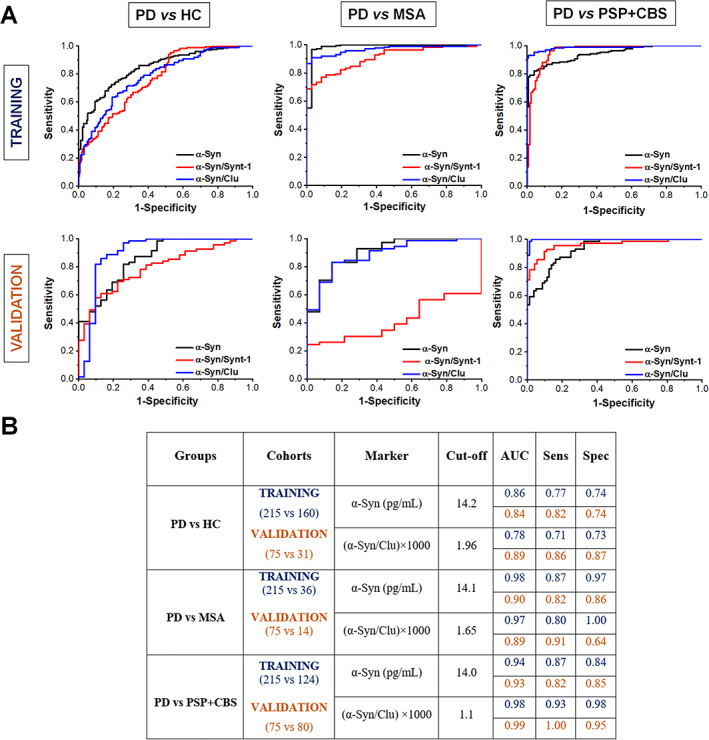
(**A**) Receiver operating characteristic (ROC) analysis was performed to evaluate the biomarker in distinguishing between PD and HC, between PD and MSA, and between PD and PSP + CBS using α‐Syn, Clu, and α‐Syn/Clu. We used a 2‐stage design model with discovery (Kiel + PROSPECT) and validation (Oxford + Brescia) cohorts to assess consistency across patient populations. (**B**) Summary of assay performance following ROC analysis across 2 groups training (blue) and validation (light brown) for each biomarker. Only the most significant (AUC > 0.80) are displayed. AUC, area under curve; Sens, sensitivity; Spec, specificity.

We then combined these measurements with our previously published data as shown in Table 1 and performed further analysis to assess the consistency and reproducibility of the assay across cohorts. We included PD patients without cognitive deficit based on a previously defined MoCA test > 21.^18^ We applied a 2‐stage design model with training and validation groups from different cohorts to robustly identify the best combination of markers that stratify parkinsonian syndromes. We found that measuring the amount of α‐synuclein in L1CAM‐immunocaptured exosomes per milliliter of serum is the most consistent biomarker in differentiating PD from MSA or PSP + CBS across cohorts, as shown in Figure 1. For PD versus MSA, we used a training group of n = 251 (ie, 215 PD vs 36 MSA subjects from Kiel + PROSPECT) to identify a cutoff value, which was then applied to an independent validation group of n = 89 subjects (ie, 75 PD vs 14 MSA from Oxford + Brescia). This showed that exosomal α‐synuclein at 14.1 pg/mL serum, exhibited a consistent performance (training vs validation), with an AUC of 0.98 versus 0.90, sensitivity of 0.87 versus 0.82, and specificity of 0.97 versus 0.86. A similar comparison between PD and HC showed an AUC of 0.86 versus 0.84, sensitivity of 0.77 versus 0.82, and specificity of 0.74 versus 0.74. For tauopathies (PSP and CBS), we used a training group of n = 339 (ie, 215 PD vs 124 PSP + CBS subjects from Kiel + PROSPECT) to identify a cutoff value, which was then applied to an independent validation group of n = 155 (ie, 75 PD vs. 80 PSP + CBS subjects from Oxford + Brescia). We found that with a cutoff at 14.0 pg/mL, exosomal α‐synuclein also differentiated PD from 4 repeat tauopathies with an AUC of 0.94 versus 0.93, sensitivity of 0.87 versus 0.82, and specificity of 0.84 versus 0.85. Because exosomal clusterin is elevated in tauopathies, the ratio of α‐synuclein to clusterin improved the diagnostic value of the test specifically in differentiating neuronal synucleinopathy from tauopathy across cohorts with an AUC of 0.98 versus 0.99, sensitivity of 0.93 versus 1, and specificity of 0.98 versus 0.95 when using a cutoff of (α‐Syn/Clu) × 1000 = 1.1. Based on our collective data in these clinically rather than neuropathologically diagnosed cohorts, a person with parkinsonian symptoms and a positive test (defined as exosomal α‐synuclein > 14.1 pg/mL) would be correctly identified as having PD in 88% of cases (positive predictive value), whereas a person with a negative test would be correctly identified in 82% of cases as having atypical parkinsonism (negative predictive value).

α‐Synuclein in L1CAM‐positive exosomes was similarly elevated in male (29.9 ± 18.7 pg/mL) and female (29.0 ± 17.5 pg/mL) participants with PD and did not correlate with UPDRS or MoCA scores (*R* ^2^ = 0.0059 and 0.0047, respectively; Figure S2), age, or disease duration (*R* ^2^ = 0.0061 and 0.0013, respectively; Figure S2). Clusterin did not correlate with UPDRS or MoCA scores in tauopathies (*R* ^2^ = 0.0022 and 0.0245, respectively; Figure S2). We also assessed whether correcting for the generic marker syntenin‐1 improved the diagnostic yield. Although the ratio of exosomal α‐synuclein/syntenin‐1 slightly improved the differentiation of PD from tauopathies (AUC, 0.96) compared with α‐synuclein, this was not seen consistently in PD versus MSA or PD versus HC.

## 3 Discussion

We performed the largest sample analysis to date to evaluate the role of α‐synuclein measurements in L1CAM‐positive exosomes as a blood‐based biomarker in PD. Our validation across 4 cohorts showed that as a single cross‐sectional measurement, a concentration of exosomal α‐synuclein above 14 pg/mL consistently distinguishes PD from MSA or PSP + CBS with an AUC of 0.90–0.98. To the best of our knowledge, our assay offers the most accurate blood‐based test to stratify patients presenting with motor symptoms into those with a neuronal synucleinopathy (ie, Lewy‐like pathology) versus patients with a glial synucleinopathy or tauopathy. Although we have not estimated the accuracy of our assay in PD and MSA cases with postmortem diagnosis, exosomal α‐synuclein outperforms total CSF α‐synuclein as a biomarker^19^, ^20^ but is currently fractionally less sensitive than CSF RT‐QuIC (sensitivity, ~95%).^21^ α‐synuclein analysis in L1CAM‐immunocaptured exosomes involves a less invasive procedure than CSF‐based measurements, and importantly, it can be multiplexed to further enhance its diagnostic accuracy. The latter is best exemplified by the improved separation of neuronal synucleinopathy from tauopathy to an AUC of 0.98–0.99 with the additional measurement of clusterin. It is noteworthy that correction for the generic exosomal cargo protein syntenin‐1 did not consistently improve the assay, which may be because of the heterogeneity in exosome biogenesis among patients or across disease states. It is currently unclear how neurodegenerative diseases influence the biogenesis or composition of subpopulations of exosomes and other extracellular vesicles.^22^, ^23^ We therefore propose measuring generic proteins to confirm successful exosome isolation rather than to correct for disease‐associated proteins.

A key technical innovation that enabled the reproducibility and improved performance of our assay is the use of poly(carboxybetaine‐methacrylate)‐coated magnetic beads, which resist nonspecific fouling to immunocapture L1CAM‐positive exosomes.^8^, ^17^, ^24^ This is important because polymer coating prevents the nonspecific binding of serum macromolecules that may interfere with exosomal L1CAM–antibody interactions on the surface of the beads and resists the nonspecific binding of free α‐synuclein, which is ~1000‐fold more abundant (ng/mL range)^25^ compared with exosome‐associated α‐synuclein (pg/mL) potentially flooding the readout.

L1CAM is primarily a neuronal antigen expressed in brain and peripheral nerves.^26^ Therefore, L1CAM‐immunocaptured exosomes most likely reflect altered processing of neuronal α‐synuclein. In this context, our finding in MSA serum samples that did not exhibit an increased α‐synuclein similar to PD raises 2 interesting possibilities: One explanation is that in MSA α‐synuclein is not released by neurons in L1CAM‐positive exosomes because it accumulates primarily in oligodendrocytes in the brain. Alternatively, MSA pathology is confined to the central nervous system, whereas Lewy body pathology also affects the enteric or other autonomic neurons early in PD.^1^, ^2^ Therefore, it is possible that α‐synuclein in L1CAM‐positive serum exosomes may arise primarily from neurons outside the brain. Either of these explanations adds credence to L1CAM‐immunocaptured exosomes as a means of developing a selective biomarker for neuronal α‐synucleinopathy.

In summary, α‐synuclein in L1CAM‐immunocaptured exosomes from serum, could be used in association with other clinical assessments to improve recruitment of patients with motor symptoms in clinical trials, especially those targeting neuronal α‐synuclein pathology.

## Author Roles

(1) Research project: A. Conception, B. Organization, C. Execution; (2) Statistical Analysis: A. Design, B. Execution, C. Review and Critique; (3) Manuscript Preparation: A. Writing of the First Draft, B. Review and Critique.

C.J.: 1A, 1B, 1C, 2A, 2B, 3A, 3B.

F.H.: 1B, 1C, 2C, 3B.

D.B.: 2C, 3B.

M.T.H.: 2C, 3B.

A.P.: 2C, 3B.

B.B.: 2C, 3B.

J.J.D.: 1C, 2C, 3B.

G.K.T.: 1A, 1B, 1C, 2A, 2B, 2C, 3A, 3B.

All authors take responsibility for the integrity of the data analysis.

## Financial Disclosures

C.J. is a senior postdoctoral fellow funded by a grant from the Selfridges Group Foundation. F.H. received grants from the German Research Council (DFG), the Thiemann Foundation, EASI‐Genomics Consortium (Horizon 2020), the Erwin‐Röver‐Foundation, and the Else Kröner‐Fresenius Foundation. D.B. served on the advisory boards of Biogen, BIAL, Lundbeck, UCB Pharma, received honoraria from AbbVie, Biogen, BIAL, Lundbeck, UCB Pharma, Zambon, Desitin, and GE, and received grants from Janssen Pharmaceutica, German Parkinson's Disease Association (dPV), Federal Ministry for Economic Affairs and Energy (BMWi), Federal Ministry of Education and Research (BMBF), EU, Parkinson Fonds Deutschland, UCB Pharma, Novartis Pharma, Lundbeck, and Damp foundation, all unrelated to the article. A.P. received speaker honoraria from BioMarin Pharmaceutical, Chiesi Pharmaceuticals, Nutricia Pharmaceuticals, UCB Pharma, and Zambon Pharmaceuticals and received travel grants from AbbVie Pharmaceuticals, BioMarin Pharmaceutical, Nutricia Pharmaceuticals, UBC Pharma, and Zambon Pharmaceuticals. B.B. reports employment with the Centre for Ageing Brain and Neurodegenerative Disorders, Neurology Unit, Department of Clinical and Experimental Sciences, University of Brescia, Brescia, Italy, and grants from the Italian Ministry of University (MURST). M.T.H. receives grants from Parkinson's UK, Oxford NIHR Biomedical Research Centre, CPT, Lab10X, NIHR, PSPA and MJFF and has received payment for advisory board attendance for Biogen, Roche, Sanofi, CuraSen Therapeutics, Evidera, and Manus Neurodynamica. J.J.D. is employed by the University of Oxford and currently receives grants from the Selfridges Group Foundation, EPSRC, and Innovate UK and received consultancy fees from Osler Diagnostics. G.K.T. received grants from the EU, MRC, EPSRC, Wellcome Trust, Selfridges Group Foundation, LAB282, Oxford NIHR Biomedical Research Centre, and MJFF and research grants from Orion, Ono, Bukwang, and Bristol‐Myers Squibb pharmaceuticals. G.T., J.D.D., and C.J. are coinventors on 2 patent applications filed in relation to the subject of this work.

## Supporting information


**FIG. S1.** Box plots of total α‐synuclein, clusterin, and syntenin‐1 from the additional 267 samples (**A–C**) analyzed in this study and our previous 468 samples (**D–F**) from patients with synucleinopathies (PD, MSA), tauopathies (PSP, CBS), or healthy controls (HC). **P < 0.01, ***P < 0.001, ****P < 0.0001. Mean values with IQR of exosomal markers and whisker range using SD with coefficient of 1 were used in the box plots.

**FIG. S2.** Pearson correlation for α‐synuclein versus UPDRS (**A**), versus MoCA (**B**), versus *a*ge (**C**) or versus disease duration (**D**) within the PD group (n = 290) and clusterin versus UPDRS (**E**) or versus MoCA (**F**) within the tauopathies (PSP + CBS, n = 204). UPDRS scores and MoCA scores were available from 78.3% and 75.9%, respectively, of all PD patients.
