# Supplementary figures and images for: Validation of α‐Synuclein in L1CAM‐Immunocaptured Exosomes as a Biomarker for the Stratification of Parkinsonian Syndromes
Source: Mov Disord. 2021 Apr 7;36(11):2663–9. doi: 10.1002/mds.28591 (PMC8663480; doi:10.1002/mds.28591)

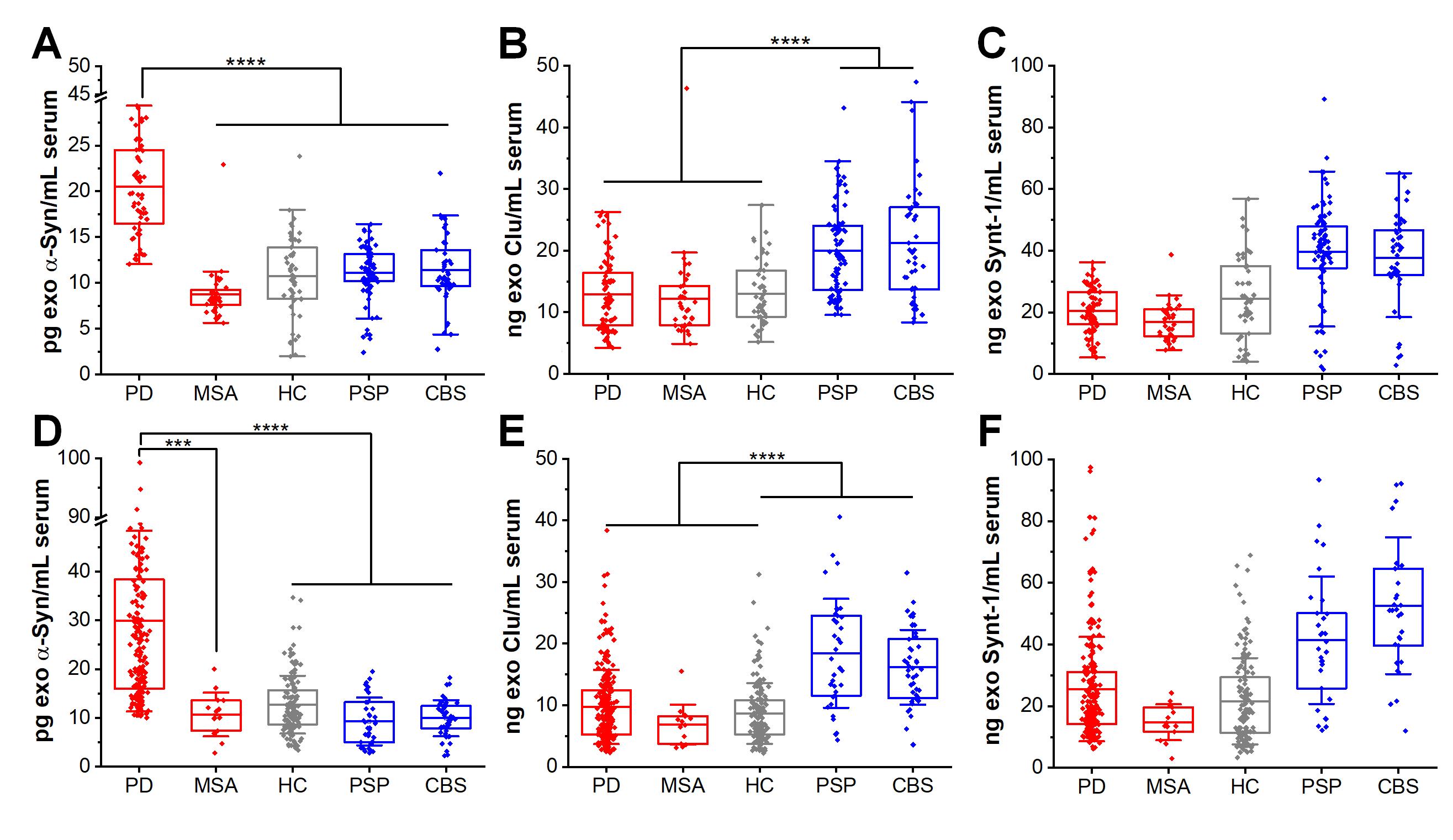

Supplement: Supplementary file 1 — FIG. S1. Box plots of total α‐synuclein, clusterin, and syntenin‐1 from the additional 267 samples (A–C) analyzed in this study and our previous 468 samples (D–F) from patients with synucleinopathies (PD, MSA), tauopathies (PSP, CBS), or healthy controls (HC). **P < 0.01, ***P < 0.001, ****P < 0.0001. Mean values with IQR of exosomal markers and whisker range using SD with coefficient of 1 were used in the box plots. [file MDS-36-2663-s001.jpg]

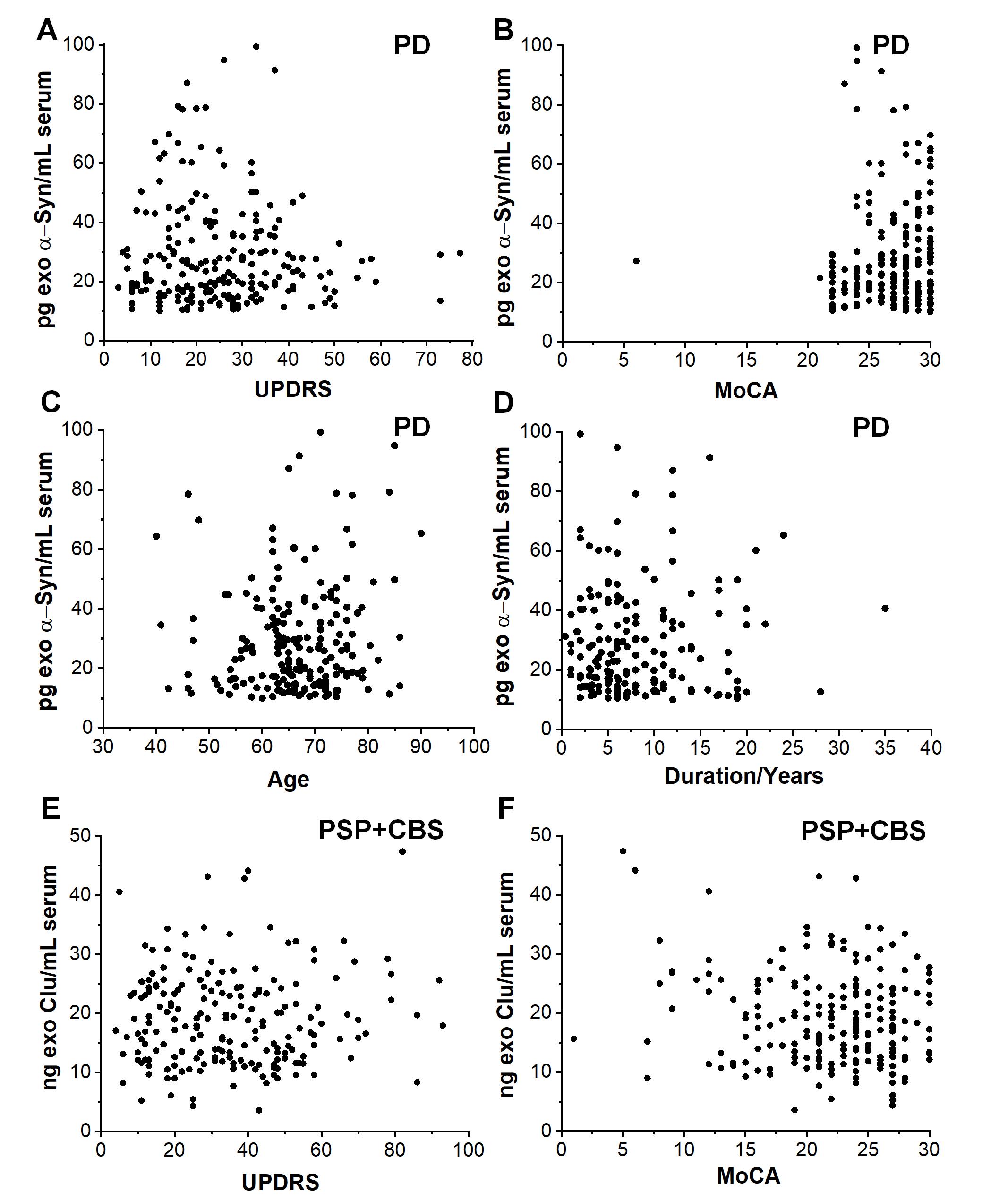

Supplement: Supplementary file 2 — FIG. S2. Pearson correlation for α‐synuclein versus UPDRS (A), versus MoCA (B), versus age (C) or versus disease duration (D) within the PD group (n = 290) and clusterin versus UPDRS (E) or versus MoCA (F) within the tauopathies (PSP + CBS, n = 204). UPDRS scores and MoCA scores were available from 78.3% and 75.9%, respectively, of all PD patients. [file MDS-36-2663-s002.jpg]
